# Factors associated with menstrual cycle irregularity and menopause

**DOI:** 10.1186/s12905-018-0528-x

**Published:** 2018-02-06

**Authors:** Jinju Bae, Susan Park, Jin-Won Kwon

**Affiliations:** College of Pharmacy and Research Institute of Pharmaceutical Sciences, Kyungpook National University, 80 Daehak-ro, Daegu, 41566 South Korea

**Keywords:** Irregular menstruation, Premature menopause, Smoking, Obesity, Stress

## Abstract

**Background:**

A regular menstrual cycle is an important indicator of a healthy reproductive system. Previous studies reported obesity, stress, and smoking as the factors that are associated with irregular menstruation and early menopause. However, the integrative effects of these modifiable risk factors have not been fully understood. This study aimed to investigate the modifiable risk factors of menstrual cycle irregularity and premature menopause, as well as their individual and combined effects among adult women in Korea.

**Method:**

This study selected adult women aged 19 years and above who had been included in the 2007–2014 Korean National Health and Nutrition Examination Survey. We used a separate dataset to analyze the risk factors of menstrual cycle irregularity and menopause (pre- and postmenopausal women: *n* = 4788 and *n* = 10,697, respectively). Univariate and multiple logistic regression analyses were conducted to evaluate the effects of smoking, drinking, obesity, and perceived level of stress on the menstrual cycle and menopause. Both logit and linear models were used in the analyses of the association between smoking and menopausal age. Equivalized household income, marital status, and educational level were considered as covariates. The modifiable risk factor scores were also calculated to integrate the effect of smoking, drinking, and obesity in the analysis.

**Result:**

Results showed that smoking status, pack-year, obesity, and perceived level of stress were significantly associated with irregular menstruation among premenopausal women. Especially, women demonstrating > 3 modifiable risk factor scores had 1.7 times higher risk of having irregular menstruation than those who had a 0 score. Meanwhile, early initiation of smoking (≤19 years) and high pack-year (≥5) were also significantly associated with premature menopause among postmenopausal women.

**Conclusion:**

This study demonstrated that modifiable risk factors, such as smoking, obesity, and stress, were significantly associated with menstrual cycle irregularity. Lifetime smoking was also correlated with early menopause. Our results suggested that healthier lifestyle practices, including, cessation of smoking, weight control, and stress management, were important factors in improving the reproductive health of women throughout life.

## Background

Menstruation is an important indicator of possible pregnancy as well as the reproductive health of women. The increasing prevalence of infertility and low fertility associated with age among women in the Korean society has generated significant interest. In fact, in 2014, the number of women who had difficulties in getting pregnant rose to 160,000, which accounted to an approximate increase of 65% over the past decade. Additionally, studies on the possible relationship between exposure to various diseases and irregular menstrual cycles or early-onset menopausal symptoms among menopausal women have been conducted [1–5].

A variety of hormones affects the menstrual cycle. Irregular menstrual cycle is a major symptom of anovulation, a phenomenon that is accompanied by decreased ovarian steroid secretion and production [6–9]. The most important cause of menstrual cycle irregularity is functional hypothalamic amenorrhea associated with decreased gonadotropin-releasing hormone secretion and hypothalamic–pituitary–adrenal (HPA) axis dysregulation [10–13]. The occurrence of these hormonal problems may lead to the development of various chronic diseases, including, infertility, heart disease, and type 2 diabetes [4, 14–16]. Moreover, the continuation of menstrual cycle irregularities occurring over long periods can result in early onset of menopause. Previous studies have shown that premature menopause increases the risk of heart disease and osteoporosis [1–3].

Irregular menstruation and early menopause may be affected by several factors, including, modifiable risk factors. Studies have shown that the changes in the female hormone levels are associated with health behaviors, obesity, and stress. For example, one study demonstrated that smoking can cause hypoestrogenism [17]. Furthermore, high stress has been shown to affect the HPA axis activity [18], whereas high BMI has been demonstrated to influence the sex hormone-binding globulin (SHBG), free androgen index (FAI), testosterone, and insulin levels [19]. Thus, previous studies have reported a significant association between lifestyle and menstruation [20–25]. However, while most of these studies on menstruation and menopause have been conducted in western countries [22], only a few have been performed in Asia, where women have relatively low BMI and smoking prevalence [26, 27]. Therefore, this study aimed to investigate the effects of modifiable risk factors on the menopausal irregularities and premature menopause in view of public health.

## Method

### Database

This study used the data of the 4th and 5th cycles (2007–2012) and the 1st and 2nd years (2013–2014) of the 6th cycle of the Korea National Health and Nutrition Examination Survey (KNHANES). The KNHANES was conducted to assess the health and nutritional status of Koreans and monitor the trends in health risk factors and prevalence of major chronic diseases to obtain data that will be used for the development and evaluation of health policies and programs in Korea. KNHANES was designed by complex, multistage, probability sampling to be representative of the civilian, non-institutionalized Korea population. In the case of 2011 survey, the sampling was carried out in two stages: i) selecting a sample of 192 primary sampling units (PSUs) in the whole country (approximately 200,000 PSUs); and ii) systematic sampling of 20 households among each PSU that consisted of an average of 60 households. All people in the selected households were eligible for the KNHANES. The data were collected from the medical check-ups and surveys on health and nutritional status. Informed consent were obtained from the participants who underwent medical check-ups, and the research scheme and data collection methods was approved by the Ethics Committee. The KNHANES data is open to the public through the website of the Korean Center for Disease Control (KCDC) after anonymization [28].

### Study participants

Although the occurrence of early menopause among the participants was examined from 2007 to 2014, the existence of menstrual cycle irregularity was assessed only from 2010 to 2012. A total of 4788 out of 13,918 women who participated in the survey from 2010 to 2012 were included in the analysis to investigate the risk factors of menstrual cycle irregularity after the exclusion of women who were aged under 19 years (*n* = 2780), did not answer the question on irregular menstrual status, or were already menopaused (*n* = 6350). To analyze the menopausal status, 10,697 out of 36,017 women included in the KNHANES survey from 2007 to 2014 were selected following the exclusion of those who were aged under 19 years (*n* = 7522) and had missing data on menopausal status or artificial menopause (*n* = 17798). Furthermore, we excluded the women who participated in the 2013–2014 KNHANES survey in the analysis of association between smoking (age at smoking initiation and pack-year) and menopause because the age of smoking initiation was not surveyed in those periods. Lastly, we included 7001 women whose menarche age data were available in the analysis of the association between menopausal and menarche ages.

**Fig. 1:**
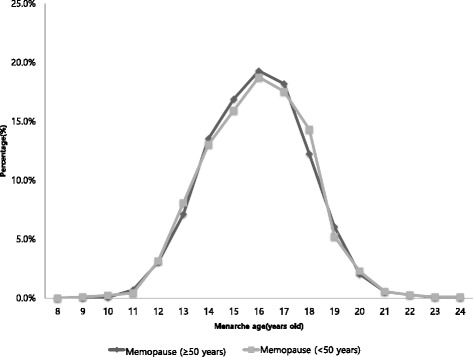
Distributions of menarche age by group of menopausal age (KNHANES, 2010–2014)

### Variables

The presence of menstrual cycle irregularity, menopausal age, and menarche age were determined using self-reported questionnaire. The question used to determine the existence of menstrual cycle irregularity was, “Do you currently have a regular menstrual cycle?” Meanwhile, the question used to identify the menarche age was, “When did you have your first menstruation?” For the question on menopausal age, the participants were initially asked whether they had amenorrhea or not. Subsequently, they were asked on the cause and age of onset of their amenorrhea. We only used the data on menopausal age of the participants who placed a check on the natural menopause category.

The participants were categorized based on their smoking status, that is, current smokers, ex-smokers, and non-smokers. Secondhand smoking was assessed using the following self-reported questionnaires: “How many hours a day are you exposed to tobacco smoke at home?” and “How many hours a day are you exposed to tobacco smoke in your workplace?” Then we categorized the total exposure time at home or in their workplaces into 3 groups as follows: no, < 1 h, and ≥1 h. Age at smoking initiation was identified using the following self-reported questionnaire: “When did you smoke a total of one cigarette?” Cigarettes per day and pack-year were counted for ex- and current smokers, including those who were daily and occasional smokers. The pack-year was calculated by multiplying the packs smoked per day (1 pack = 20 cigarettes) by years as a smoker. Additionally, the participants were divided into the following five groups based on their frequency of alcohol consumption: 0, ≤1 time a month, 2–4 times a month, 2–3 times a week, and ≥4 times a week. The participants were also categorized into the following five groups based on their BMI and in accordance with the Asian obesity guideline: underweight (0 to < 18.5), normal weight (18.5 to < 23), overweight (23 to < 25), obese I (25 to < 30), and obese II (≥30). Moreover, the participants were asked on their perceived level of stress using the question “How much do you feel stress generally in your daily life?” with responses such as “few,” “a little,” “much,” and “very much.” Meanwhile, the area of residence of the participants was categorized into rural or urban area based on the administrative district. The equivalized household income was calculated by dividing the household income with the square root of the number of household members; subsequently, the income was grouped into quartiles. The participants were also categorized based on their marital status, that is, non-married or married. Furthermore, they were grouped into four based on their educational level: elementary undergraduate or middle school, high school, and college or university graduate. The participants who were unable to graduate were categorized into the lower educational level group.

Modifiable risk factor scores were assigned for the integrated variables, including smoking status, BMI, and alcohol consumption frequency. The scores were given as follows: 0 point each for non-smokers, BMI ranges from underweight to overweight (0 to < 25 BMI), and nondrinkers; 1 point each for ex-smokers, obesity I (25 to < 30), and 1–4 times per month alcohol consumption frequency; and 2 points each current smoker, obesity II (≥30 BMI), and ≥ 2 times a week alcohol consumption frequency. Subsequently, we summed the given scores and categorized these scores into four groups (0, 1, 2, ≥3).

### Statistical analysis

For the descriptive statistics, the categorical data were presented as percentage, whereas the continuous data were presented as mean and standard deviation (SD). We applied the weight for all analysis to ensure representativeness of the Korean population. For example, unweighted frequencies and weighted proportions with standard errors (SE) were presented for categorical data. Univariate and multiple logistic analyses were conducted to determine the effect of the risk factors (i.e. perceived level of stress, lifetime smoking status, smoking status regardless of exposure to secondhand smoke, smoking status including exposure to secondhand smoke, alcohol, obesity, residence, income, marital status, and educational level) on menstrual cycle irregularity. Additionally, the effect of smoking on early menopause was investigated using univariate logistic or linear regression analysis. The survey procedure was incorporated into the logistic regression analysis so that the complex survey design and sample weight will be reflected. All the data were analyzed using the SAS program (ver. 9.4; SAS Institute, Cary, NC, USA).

## Result

The average age of women with both regular and irregular menstrual cycles was 36.4 (SD, ±8.5) and 37.0 (SD, ±11.1) years, respectively. Table 1 presents the characteristics of the participants with and without regular menstrual cycles.

**Table 1:** Descriptive characteristics of premenopausal women by menstrual cycle regularity (KNHANES, 2010–2012)

	Total (*n* = 4788)			Regular (*n* = 4103)			Irregular (*n* = 685)		
	n	%	SE	n	%	SE	n	%	SE
Age, years									
19 to < 30	1157	30.6	1.0	943	28.9	1.0	214	39.7	2.3
30 to <40	1737	31.6	0.9	1568	33.4	1.0	169	21.6	1.8
≥ 40	1894	37.8	0.9	1592	37.7	1.0	302	38.7	2.1
Residence									
Rural	601	13.8	1.4	504	13.4	1.4	588	15.7	2.3
Urban	4187	86.2	1.4	3599	86.6	1.4	97	84.3	2.3
Household income, quartiles									
I	1130	27.7	0.9	968	27.7	1.0	162	27.5	2.3
II	1171	24.7	0.8	1024	25.4	0.9	147	21.2	1.9
III	1203	23.9	0.8	1009	23.2	0.9	194	27.4	2.2
IV	1236	22.6	0.9	1060	22.6	0.9	176	22.6	2.0
Missing data	48	1.1	0.2	42	1.1	0.2	6	1.2	0.7
Marital status									
Married	3563	69.2	1.0	3090	70.6	1.0	473	61.4	2.2
Not married	1225	30.8	1.0	1013	29.4	1.0	212	38.6	2.2
Educational level									
Elementary school and below	144	3.2	0.3	104	2.8	0.3	40	5.9	1.0
Middle school	251	5.8	0.4	186	5.1	0.4	65	9.6	1.3
High school	2183	48.4	1.0	1848	47.6	1.1	335	53.0	2.4
University and above	2202	42.3	1.0	1959	44.3	1.1	243	31.2	2.2
Missing data	8	0.2	0.1	6	0.2	0.1	2	0.4	0.3
Smoking status									
Non-smoker	4264	87.2	0.6	3663	87.6	0.6	601	85.2	1.6
Ex-smoker	208	4.8	0.4	181	4.9	0.4	27	4.5	1.0
Current smoker	316	8.0	0.5	259	7.6	0.5	57	10.3	1.5
Secondhand smoking									
None	1359	27.3	0.8	1174	27.6	0.9	185	25.3	2.0
< 1 h	211	4.8	0.4	183	4.9	0.4	28	4.5	1.1
≥ 1 h	392	9.8	0.6	334	9.9	0.6	58	9.1	1.3
Missing data	2826	58.1	0.9	2412	57.6	1.0	414	61.1	2.2
Cigarettes per day									
Non-smoker	4264	87.2	0.6	3663	87.6	0.6	601	85.2	1.6
< 20 cigarettes	478	11.7	0.6	400	11.3	0.6	78	13.7	1.6
≥ 20 cigarettes	44	1.1	0.2	38	1.1	0.2	6	1.1	0.5
Missing data	2	0.0	0.0	2	0.0	0.0			
Age at smoking initiation									
Non-smoker	4264	87.2	0.6	3663	87.6	0.6	601	85.2	1.6
< 20 years	242	6.5	0.5	199	6.2	0.5	43	8.1	1.3
≥ 20 years	282	6.3	0.4	241	6.2	0.5	41	6.7	1.2
Pack-year									
Non-smoker	4264	87.2	0.6	3663	87.6	0.6	601	85.2	1.6
Above the median (2.5 pack-years)	249	6.0	0.4	206	5.6	0.4	43	8.0	1.3
Below or equal to the median (2.5 pack-years)	257	6.4	0.5	220	6.3	0.5	37	6.4	1.2
Missing data	18	0.4	0.1	14	0.4	0.1	4	0.4	0.2
Alcohol consumption									
None	604	12.0	0.6	520	12.1	0.6	84	11.5	1.5
≤ 1 time per month	2085	43.2	0.9	1783	42.7	0.9	302	45.9	2.1
2–4 times a month	1164	24.6	0.8	1003	25.2	0.8	161	21.2	1.9
2–3 times a week	456	9.7	0.6	396	9.9	0.6	60	8.8	1.3
≥ 4 times a week	100	2.5	0.3	81	2.3	0.3	19	3.4	1.0
Missing data	379	8.0	0.5	320	7.8	0.6	59	9.2	1.4
Body mass index									
0 to < 18.5 kg/m^2^	443	10.0	0.5	388	10.2	0.6	55	9.3	1.3
18.5 to < 23 kg/m^2^	2521	51.0	1.0	2211	52.2	1.0	310	44.8	2.4
23 to < 25 kg/m^2^	807	17.0	0.6	689	17.1	0.7	118	16.0	1.6
25 to < 30 kg/m^2^	811	17.2	0.7	662	16.3	0.8	149	22.2	1.8
≥ 30 kg/m^2^	191	4.5	0.4	143	4.0	0.4	48	7.3	1.3
Missing data	15	0.3	0.1	10	0.3	0.1	5	0.5	0.2
Perceived stress									
Little	396	8.0	0.5	347	8.2	0.5	49	7.1	1.2
A little	2847	58.3	0.9	2477	59.3	0.9	370	52.8	2.3
Much	1304	28.2	0.8	1086	27.4	0.9	218	32.1	2.1
Very much	241	5.5	0.4	193	5.0	0.4	48	8.0	1.2
Modifiable risk factor score^a^									
0	450	9.38	0.52	399	9.8	0.58	51	7.04	1.24
1	2452	54.6	0.97	2134	55.5	1.02	318	49.8	2.48
2	925	21.3	0.77	787	21.1	0.86	138	22.4	1.92
≥ 3	568	14.72	0.7	454	13.7	0.7	114	20.7	1.99

^a^The given scores were summed and categorized based on smoking status, obesity level, and drinking frequency. The given score of each variable was as follows: 0 point each for non-smokers, underweight to overweight  (0 to < 25 BMI), and nondrinkers; 1 point each for ex-smokers, obesity I (25 to < 30), and 1–4 times per month alcohol consumption frequency; and 2 points each current smoker, obesity II (≥30 BMI), and ≥ 2 times a week alcohol consumption frequency

Table 2 shows the factors affecting the menstrual cycle of premenopausal women. In the univariate analysis, age, smoking status, pack-year, body weight, alcohol, perceived level of stress, marital status, educational level, and modifiable risk factor scores were associated with irregular menstruation (Table 2). Pack-year did not show a dose–response relationship with irregular menstruation. The pack-year with the lower level from the median value was associated with higher odds ratios (ORs) of menstrual cycle irregularity than those on the upper level. Based on the results of multiple logistic regression analysis involving obesity and smoking status, obese women (≥25 BMI) showed higher risk of irregular menstruation than normal-weight women (25- < 30 kg/m^2^ OR = 1.56, 95% CI = 1.19–2.03; ≥30 kg/m^2^, OR = 1.81, 95% CI = 1.17–2.79). In the multiple logistic regression analysis that included age, behavior score, marital status, perceived stress, and educational level, ≥3 modifiable risk factor score was significantly associated with menstrual cycle irregularity. Following the adjustments of modifiable risk factor scores, perceived stress and educational level were significantly associated with menstrual cycle irregularity. Women who responded their perceived stress as ‘very much’ had 1.74 times higher OR of menstrual cycle irregularity than women who did as ‘little’. As educational level was lowered, the risk of menstrual cycle irregularity increased (elementary school and below (reference = high school), OR = 2.07, 95% CI = 1.24–3.46).

**Table 2:** Factors affecting the menstrual cycle irregularity of premenopausal women (KNHANES, 2010-2012)

	Univariate logistic (*n* = 4788)			Multivariable (*n* = 4773)			Multivariable (*n* = 4388)		
	OR	95% CI		OR	95% CI		OR	95% CI	
Age, years									
19 to < 30	1.00			1.00			1.00		
30 to <40	0.47	0.37	0.61	0.60	0.41	0.90	0.63	0.41	0.95
≥ 40	0.75	0.60	0.93	0.79	0.52	1.18	0.84	0.55	1.28
Residence									
Rural	1.00								
Urban	0.83	0.62	1.12						
Household income, quartiles									
I	1.00								
II	0.84	0.63	1.12						
III	1.19	0.89	1.59						
IV	1.01	0.76	1.35						
Marital status									
Married	1.00			1.00			1.00		
Not married	1.51	1.24	1.85	1.46	1.00	2.14	1.73	1.38	2.16
Educational level									
Elementary school and below	1.91	1.24	2.94	2.04	1.28	3.25	2.07	1.24	3.46
Middle school	1.69	1.18	2.42	1.81	1.23	2.66	1.79	1.20	2.65
High school	1.00			1.00			1.00		
University and above	0.63	0.50	0.79	0.65	0.51	0.82	0.60	0.47	0.76
Smoking status									
Non-smoker	1.00								
Ex-smoker	0.95	0.59	1.54	0.97	0.58	1.60			
Current smoker	1.40	1.01	1.94	1.11	0.79	1.56			
Secondhand smoking									
None	1.00								
< 1 h	0.99	0.58	1.69						
≥ 1 h	1.00	0.69	1.43						
Cigarettes per day									
Non-smoker	1.00								
< 20 cigarettes	1.24	0.93	1.65						
≥ 20 cigarettes	1.10	0.45	2.71						
Age at smoking initiation									
Non-smoker	1.00								
< 20 years	1.35	0.93	1.97						
≥ 20 years	1.10	0.74	1.64						
Pack-year									
Non-smoker	1.00								
Above the median (2.5 pack-years)	1.45	1.01	2.10						
Below or equal to the median (2.5 pack-years)	1.04	0.67	1.61						
Alcohol consumption									
None	1.00								
≤ 1 time per month	1.13	0.83	1.54						
2–4 times a month	0.89	0.62	1.26						
2–3 times a week	0.94	0.60	1.46						
≥ 4 times a week	1.58	0.78	3.20						
Body mass index									
0 to < 18.5 kg/m^2^	1.06	0.73	1.55	0.92	0.62	1.36			
18.5 to < 23 kg/m^2^	1.00			1.00					
23 to < 25 kg/m^2^	1.09	0.82	1.43	1.11	0.84	1.49			
25 to < 30 kg/m^2^	1.58	1.23	2.04	1.56	1.19	2.03			
≥ 30 kg/m^2^	2.14	1.42	3.23	1.81	1.17	2.79			
Perceived stress									
Little	1.00			1.00			1.00		
A little	1.02	0.70	1.50	1.07	0.73	1.57	1.09	0.72	1.65
Much	1.34	0.90	2.00	1.31	0.89	1.95	1.28	0.83	1.98
Very much	1.83	1.12	3.01	1.73	1.05	2.86	1.74	1.02	2.99
Modifiable risk factor score^a^									
0							1.00		
1							1.13	0.75	1.70
2							1.30	0.83	2.05
≥ 3							1.61	1.03	2.52

*OR* odds ratios, *CI* confidence interval

^a^The given scores were summed and categorized based on smoking status, obesity level, and drinking frequency. The given score of each variable was as follows: 0 point each for non-smokers, underweight to overweight (0 to < 25 BMI), and nondrinkers; 1 point each for ex-smokers, obesity I (25 to < 30), and 1–4 times per month alcohol consumption frequency; and 2 points each current smoker, obesity II (≥30 BMI), and ≥ 2 times a week alcohol consumption frequency

Table 3 displays the association between smoking and early menopause. To assess the effect of smoking on the menopausal status, we divided the population into two groups: early menopause (menopause before 50 years old) and late menopause (menopause at 50 years old and above) groups. The average ages (±SD) of the late and early menopause groups were 52.6 ± 2.4 and 45.5 ± 3.8 years, respectively. The ex- and current smokers had 1.34 and 1.68 times higher ORs than the non-smokers. Specifically, the ex- and current smokers had had higher ORs in terms of the number of cigarettes smoked per day, age at smoking initiation, total pack-year, and pack-year before menopause than the non-smokers, although a dose–response relationship was not observed. Furthermore, the current smokers had significantly lower mean menopausal age than the ex- and non-smokers, based on the result of the regression analysis. The current smokers had approximately 1.14 (SE = 0.283) lower average menopausal age than the non-smokers.

**Table 3:** Effect of smoking on menopause among postmenopausal women (KNHANES, 2007–2014)

Variables	Early menopause (< 50 years)		Late menopause (≥50 years)		Logistic regression for early menopause (< 50 years)^a^			Linear regression for menopausal age (categorical variable)^a^			Linear regression for menopausal age (continuous variable)^a^		
	n	%	n	%	OR	95% CI		*β*	SE	*p*-value	*β*	SE	*p*-value
	(mean)	(SD)	(mean)	(SD)									
Total	4930	45.9	5767	54.1									
Current age, mean (SD)	64.5	0.2	62.8	0.2									
Average menopausal age, mean (SD)	45.6	0.1	52.5	0.0									
Smoking status													
Non-smoker	4447	90.6	5343	93.6	1.00			1.000					
Ex-smoker	172	3.7	160	2.9	1.34	1.02	1.77	−0.615	0.365	0.093			
Current smoker	236	5.7	175	3.5	1.68	1.30	2.17	−1.137	0.283	<.0001			
Total	4855	100.0	5678	100.0									
Cigarettes per day													
Non-smoker	4447	92.3	5343	95.1	1.00			1.000					
< 20 cigarettes	268	6.1	205	3.9	1.61	1.26	2.07	−1.104	0.302	0.000			
≥ 20 cigarettes	69	1.5	55	1.0	1.53	1.00	2.33	−0.486	0.444	0.274			
Total	4784	100.0	5603	100.0							−0.038	0.019	0.046
Age at smoking initiation^b^													
Non-smoker	3409	89.9	3993	93.1	1.00			1.000					
< 20 years	36	1.2	15	0.4	2.93	1.45	5.90	−1.438	0.461	0.002			
≥ 20 years	298	8.9	246	6.4	1.43	1.13	1.82	−0.827	0.293	0.005			
Total	3743	100.0	4254	100.0							−0.004	0.001	<.0001
Pack-year (total)^b^													
Non-smoker	3409	90.2	3993	93.4	1.00								
Below or equal to the median (≤9.8 pack-years)	153	4.9	175	3.4	1.50	1.11	2.01	−0.773	0.342	0.024			
Above the median (>9.8 pack-years)	172	4.9	78	3.3	1.57	1.17	2.09	−1.037	0.389	0.008			
Total	3734	100.0	4246	100.0							−0.025	0.015	0.103
Pack-year (before menopause)^b^													
Non-smoker	3409	92.0	3993	93.7	1.00			1.000					
Below or equal to the median (≤5 pack-years)	133	4.0	107	2.7	1.54	1.11	2.13	−1.076	0.370	0.004			
Above the median (>5 pack-years)	123	4.0	131	3.6	1.14	0.83	1.57	0.417	0.340	0.220			
Total	3665	100.0	4231	100.0							0.035	0.020	0.077

*OR* odds ratios, *CI* confidence interval; ^a^ Univariate model ^ b^ Excluded KNHANES 2013-2014 because age at smoking initiation was not surveyed

Figure 1 shows the proportions of each menarche age by groups of early (< 50 years) and late menopause (≥50 years). There was no difference in the menarche ages of the early and late menopause groups; thus, early menarche age was not a factor to accelerate the early menopause in our subjects.

## Discussion

Our study investigated the various factors influencing the menstrual cycle and the effects of smoking on menopausal onset. In our study, the risk factors for menstrual cycle irregularity were perceived stress, obesity, smoking, and marital status. Furthermore, early initiation of smoking and high cigarette consumption were significantly associated with premature menopause based on the results of analyses using both logit and linear regression models. This study focused on modifiable risk factors, such as smoking, obesity, and perceived stress, and revealed the importance of these factors in the improvement of women’s health.

With regard to stress, our study showed that high level of perceived stress was associated with high probability of menstrual cycle irregularity. A previous study conducted among 696 women aged 20–40 years revealed that menstrual cycle irregularity was associated with high chronic stress level [29]. Moreover, past studies that utilized special tools to check the stress level (i.e., > 20 in the Perceived Stress Scale or Global Severity Index) of university students also indicated that stress was correlated with irregular menstruation [30, 31]. One theory that explained the mechanism through which stress affects the menstrual cycle involves the HPA axis. This theory suggests that a reduction in the HPA axis activity leads to the occurrence of menopause. When the stress level is high, the HPA axis activity is interrupted. Thus, women who are suffering from considerable stress may experience more irregularities in menstruation than those who are not under stress [32].

In the present study, women who had BMI of 25–30 or ≥30 have high possibility of developing irregular menstruation. This finding is also consistent with those of previous studies on the association between irregular menstruation and obesity. For instance, one study that involved 14,779 women who were 19–23 years old showed that those with BMI of < 17, 17–18.5, 18.5–20, 25–30, and > 30 had 1.6, 1.2, 1.2, 1.1, and 1.4 times higher probability of developing menstrual cycle irregularity, respectively, than those with BMI of 20–25 BMI [33]. Furthermore, another study conducted among 1095 female students in childbearing years (18–25 years old) in Taiwan demonstrated that women with BMI of > 27 had 18.48 times higher risk of developing menstrual cycle irregularity than those with BMI of 18.5–23.9 [20]. Additionally, a study on irregular menstruation, which was defined as > 15 days of menstrual cycle difference, that was conducted among 726 women showed that women with BMI of > 30 had 2.61 times higher risk of developing irregular menstruation than those with BMI of 25–29.9 [22]. The mechanism behind the association between BMI and irregular menstruation may be attributed to low SHBG level and high testosterone and FAI levels. The present study showed that women who had higher BMI developed irregular menstruation caused by low SHBG level and high testosterone, FAI, and insulin levels. An increase in the BMI resulted to changes (either decrease or increase) in the value of SHBG (− 0.44), testosterone (+ 0.17), FAI (+ 0.42), and insulin (+ 0.54) [22].

The univariate regression analysis revealed that smoking was correlated with irregular menstruation; however, this association was not found in the multivariable regression analysis. To determine the effect of smoking on menstrual cycle irregularity, we categorized the smoking-related factors into the following: lifetime smoking status, secondhand smoking, cigarettes per day, age at smoking initiation, and pack-year. The results of the univariate logistic regression analysis revealed a statistically significant association between smoking status and irregular menstruation. Specifically, the current smokers had 1.4 times higher prevalence of menstrual cycle irregularity than non-smokers. However, the multivariable logistic regression analysis did not show a statistically significant difference in the prevalence of menstrual cycle irregularity between non-smokers and current smokers. This difference may be explained by multicollinearity because various factors, such as perceived stress, obesity, and alcohol consumption, were correlated with smoking.

The univariate analysis also revealed an association between menopause and smoking, although a dose–response relationship with regard to the pack-year was not observed. Participants who smoked at younger and older age had a tendency to develop early menopause. A previous study conducted among 14,889 women who were 18–23 years old in Austria indicated that ex-smokers had 1.2 times higher risk of developing irregular menstruation than non-smokers. For current smokers, the higher the number of smoked cigarettes, the higher the risk to develop irregular menstruation [33]. The findings of several previous studies also supported the association between smoking and early menopause. For example, one study involving 543 participants showed that current smokers were 0.8 years younger than non-smokers; however, no statistically significant difference was observed in regard to secondhand smoking between ex-smokers and current smokers [34]. Additionally, another previous study conducted among women aged 45–55 years old in Massachusetts demonstrated that smokers experienced menopause was 1.8 years earlier than non-smokers [35].

Additionally, a study based on the US National Health and Nutrition Examination Survey III demonstrated that smoking elevated the risk of developing early menopause among 5029 women who were older than 25 years old [36]. A systematic review of 96 articles based on 109 studies reported a association between smoking and early menopause, although a clear association between the quantities of cigarettes smoked or smoking period was not observed [21]. Moreover, one study also analyzed the relationship between prenatal and childhood household smoke exposure or adult active smoking and early natural menopause. The results showed current smokers who had been smoking period for > 26 years or > 10 cigarettes/day had higher risk of developing early natural menopause than the other current smokers [37].

The association between smoking and menopause may be attributed to the development of a hypoestrogenic state that is induced by smoking. A study conducted from 1977 to 1984 among 5000 women who were 34 years old or above showed that smokers had 19% lower level of estradiol based on urine test than non-smokers after menopause [38]. Furthermore, a study that involved 603 premenopausal women demonstrated that current smokers had lower total estrogen metabolite (EM) levels than non-smokers. These participants were shown to have significantly lower levels of parent estrogens, such as estradiol, 1-methoxyestradiol (a metabolite of the 2-hydroxylation pathway), estriol (an EM of the 16-hydroxylation pathway), and 16-epiestriol [39]. Moreover, one study conducted from 1982 to 1984 among 350 women aged 45–69 years revealed that smokers use 1.46 times more hormonal replacement therapy (HRT) than non-smokers (OR = 1.46, *p* = 0.005). On the other hand, the use of HRT was not statistically significant among ex-smokers. HRT is used when the estrogen level becomes low. Therefore, the increase use of HRT among smokers than non-smokers indicated that smoking induces hypoestrogenic state [40]. Another mechanism through which smoking induces hypoestrogenic state involves the increase in the level of HPA axis hormones, including adrenocorticotropic hormone, cortisol, and dehydroepiandrosterone, such as during stress [41].

The association between alcohol consumption and menstrual cycle irregularity was not found to be statistically significant. However, when we considered the behavioral patterns such as smoking status, obesity level, and alcohol consumption, these negative behaviors were significantly associated with the increased prevalence of menstrual cycle irregularity. While, smoking was inversely related to obesity, alcohol consumption could result in increasing the prevalence of obesity. Therefore, it may be plausible to consider the integration of behaviors when investigating menstrual cycle irregularity.

Menarche age was not significantly different between women who attained menopause before or after the age of 50 years. Some debates still exist on the relationship between menarche and menopausal ages. Women who began their menstruation at the age of 11 years or younger have an 80% higher chance of having menopause at the age of 40 years than those who started menstruating at the age of 13 years. Additionally, the risk of having menopause at the age of 40–44 years was approximately 30% [42]. However, our study indicated that premature menopause may be caused by acquired and environmental factors, rather than the menarche age. Hence, further research on this topic would be necessary [43, 44].

Our study has several limitations to consider when interpreting the results. First, because our study used cross-sectional data, causal relationships could not be determined. Second, most data on the participants’ characteristics were collected through self-reported questionnaires, except for BMI. Third, when assessing the factors affecting menopause, we could not consider other factors, except for smoking, because information regarding the participants’ at the time of their menopausal age was not available.

## Conclusion

Our study results showed the importance of healthier behavioral practices to maintain menstrual cycle regularity, especially when considering that smoking may be associated with the occurrence of early menopause. Given the association between early menopause or irregular menstruation and women’s health, improvements in health behaviors should be emphasized in view of public health.

## References

[1] Rahman I, Akesson A, Wolk A (2015). Relationship between age at natural menopause and risk of heart failure. Menopause.

[2] Atsma F, M-LE B, Grobbee DE, van der Schouw YT (2006). Postmenopausal status and early menopause as independent risk factors for cardiovascular disease: a meta-analysis. Menopause.

[3] Gallagher JC (2007). Effect of early menopause on bone mineral density and fractures. Menopause.

[4] Solomon CG, Hu FB, Dunaif A, Rich-Edwards J, Willett WC, Hunter DJ (2001). Long or highly irregular menstrual cycles as a marker for risk of type 2 diabetes mellitus. JAMA.

[5] Solomon CG, Hu FB, Dunaif A, Rich-Edwards JE, Stampfer MJ, Willett WC (2002). Menstrual cycle irregularity and risk for future cardiovascular disease. J Clin Endocrinol Metab.

[6] Kato I, Toniolo P, Koenig KL, Shore RE, Zeleniuch-Jacquotte A, Akhmedkhanov A (1999). Epidemiologic correlates with menstrual cycle length in middle aged women. Eur J Epidemiol.

[7] Waller K, Swan SH, Windham GC, Fenster L, Elkin EP, Lasley BL (1998). Use of urine biomarkers to evaluate menstrual function in healthy premenopausal women. Am J Epidemiol.

[8] Van Voorhis BJ, Santoro N, Harlow S, Crawford SL, Randolph J (2008). The relationship of bleeding patterns to daily reproductive hormones in women approaching menopause. Obstet Gynecol.

[9] Mumford SL, Steiner AZ, Pollack AZ, Perkins NJ, Filiberto AC, Albert PS (2012). The utility of menstrual cycle length as an indicator of cumulative hormonal exposure. J Clin Endocrinol Metab.

[10] Reindollar RH, Novak M, Tho SP, McDonough PG (1986). Adult-onset amenorrhea: a study of 262 patients. Am J Obstet Gynecol.

[11] Loucks AB, Thuma JR (2003). Luteinizing hormone pulsatility is disrupted at a threshold of energy availability in regularly menstruating women. J Clin Endocrinol Metab.

[12] Liu JH (1990). Hypothalamic amenorrhea: clinical perspectives, pathophysiology, and management. Am J Obstet Gynecol.

[13] Berga S, Naftolin F (2012). Neuroendocrine control of ovulation. Gynecol Endocrinol.

[14] Cooper GS, Ephross SA, Weinberg CR, Baird DD, Whelan EA, Sandler DP (1999). Menstrual and reproductive risk factors for ischemic heart disease. Epidemiology.

[15] Friday KE, Dong C, Fontenot RU (2001). Conjugated equine estrogen improves glycemic control and blood lipoproteins in postmenopausal women with type 2 diabetes. J Clin Endocrinol Metab.

[16] La Vecchia C, Decarli A, Franceschi S, Gentile A, Negri E, Parazzini F (1987). Menstrual and reproductive factors and the risk of myocardial infarction in women under fifty-five years of age. Am J Obstet Gynecol.

[17] Westhoff C, Gentile G, Lee J, Zacur H, Helbig D (1996). Predictors of ovarian steroid secretion in reproductive-age women. Am J Epidemiol.

[18] Maniam J, Antoniadis C, Morris MJ (2014). Early-life stress, HPA axis adaptation, and mechanisms contributing to later health outcomes. Front Eendocrinol.

[19] Freeman EW, Sammel MD, Lin H, Gracia CR (2010). Obesity and Reproductive hormone levels in the transition to menopause. Menopause.

[20] Chang PJ, Chen PC, Hsieh CJ, Chiu LT (2009). Risk factors on the menstrual cycle of healthy Taiwanese college nursing students. Aust N Z J Obstet Gynaecol.

[21] Parente RC, Faerstein E, Celeste RK, Werneck GL (2008). The relationship between smoking and age at the menopause: a systematic review. Maturitas.

[22] Wei S, Schmidt MD, Dwyer T, Norman RJ, Venn AJ (2009). Obesity and menstrual irregularity: associations with SHBG, testosterone, and insulin. Obesity.

[23] Ju H, Jones M, Mishra GD (2015). A U-shaped relationship between body mass index and dysmenorrhea: a longitudinal study. PLoS One.

[24] Dars S, Sayed K, Relationship YZ (2014). Of menstrual irregularities to BMI and nutritional status in adolescent girls. Pak J Med Sci.

[25] Lee SS, Kim DH, Nam G-E, Nam H-Y, Kim YE, Lee SH (2016). Association between metabolic syndrome and menstrual irregularity in middle-aged Korean women. Korean J Fam Med.

[26] James PT, Leach R, Kalamara E, Shayeghi M (2001). The worldwide obesity epidemic. Obes Res.

[27] Ng M, Freeman MK, Fleming TD, Robinson M, Dwyer-Lindgren L, Thomson B (2014). Smoking prevalence and cigarette consumption in 187 countries, 1980-2012. JAMA.

[28] Kweon S, Kim Y, M-j J, Kim Y, Kim K, Choi S (2014). Data resource profile: the Korea National Health and nutrition examination survey (KNHANES). Int J Epidemiol.

[29] Palm-Fischbacher S, Ehlert U (2014). Dispositional resilience as a moderator of the relationship between chronic stress and irregular menstrual cycle. J Psychosom Obstet Gynaecol.

[30] Nagma S, Kapoor G, Bharti R, Batra A, Batra A, Aggarwal A (2015). To evaluate the effect of perceived stress on menstrual function. J Clin Diagn Res.

[31] Delara M, Woodgate RL (2015). Psychological distress and its correlates among university students: a cross-sectional study. J Pediatr Adolesc Gynecol.

[32] Kalantaridou SN, Makrigiannakis A, Zoumakis E, Chrousos GP (2004). Stress and the female reproductive system. J Reprod Immunol.

[33] Mishra GD, Dobson AJ, Schofield MJ (2000). Cigarette smoking, menstrual symptoms and miscarriage among young women. Aust N Z J Public Health.

[34] Cooper GS, Sandler DP, Bohlig M (1999). Active and passive smoking and the occurrence of natural menopause. Epidemiology.

[35] Mckinlay SM, Brambilla DJ, Posner JG (2008). “Reprint of” the normal menopause transition. Maturitas.

[36] Fleming LE, Levis S, LeBlanc WG, Dietz NA, Arheart KL, Wilkinson JD (2008). Earlier age at menopause, work and tobacco smoke exposure. Menopause.

[37] Tawfik H, Kline J, Jacobson J, Tehranifar P, Protacio A, Flom JD (2015). Life course exposure to smoke and early menopause and menopausal transition. Menopause.

[38] MacMahon B, Trichopoulos D, Cole P, Brown J (1982). Cigarette smoking and urinary estrogens. N Engl J Med.

[39] Gu F, Caporaso NE, Schairer C, Fortner RT, Xu X, Hankinson SE (2013). Urinary concentrations of estrogens and estrogen metabolites and smoking in caucasian women. Cancer Epidemiol Biomark Prev.

[40] Greenberg G, Thompson S, Meade T (1987). Relation between cigarette smoking and use of hormonal replacement therapy for menopausal symptoms. J Epidemiol Community Health.

[41] Goletiani NV, Siegel AJ, Lukas SE, Hudson JI (2015). The effects of smoked nicotine on measures of subjective states and hypothalamic-pituitary-adrenal axis hormones in women during the follicular and luteal phases of the menstrual cycle. J Addict Med.

[42] Mishra G, Pandeya N, Dobson A, Chung H, Anderson D, Kuh D (2017). Early menarche, Nulliparity, and the risk for premature and early natural menopause. Hum Reprod.

[43] Gold EB (2011). The timing of the age at which natural menopause occurs. Obstet Gynecol Clin N Am.

[44] Gold EB, Bromberger J, Crawford S, Samuels S, Greendale GA, Harlow SD (2001). Factors associated with age at natural menopause in a multiethnic sample of midlife women. Am J Epidemiol.

